# UiO‐66 metal–organic frameworks in biomedicine: From structural tunability to bioimaging, photodiagnostics, and photodynamic cancer therapy

**DOI:** 10.1002/2211-5463.70266

**Published:** 2026-05-04

**Authors:** Veronika Huntošová, Grigorii Rakhalskii, Miroslav Almáši

**Affiliations:** ^1^ Center for Interdisciplinary Biosciences, Technology and Innovation Park P. J. Šafárik University in Košice Slovak Republic; ^2^ Institute of Animal Biochemistry and Genetics, Centre of Biosciences Slovak Academy of Sciences Bratislava Slovak Republic; ^3^ Department of Biophysics, Faculty of Science P. J. Šafárik University in Košice Slovak Republic; ^4^ Department of Inorganic Chemistry, Faculty of Science P. J. Šafárik University in Košice Slovak Republic

**Keywords:** bioimaging, metal–organic frameworks, photodynamic therapy, targeted nanomedicine, UiO‐66

## Abstract

UiO‐66‐type zirconium metal–organic frameworks (MOFs) have emerged as robust and highly tunable nanoplatforms for biomedical applications owing to their permanent porosity, exceptional chemical stability, and versatile functionalization pathways. Here, we summarize recent advances in engineering UiO‐66‐based nanoparticles for drug delivery, multimodal bioimaging, photodiagnostics, and photodynamic therapy (PDT). Precise control over composition, surface chemistry, and postsynthetic modifications allow for high drug loading, stimuli‐responsive release, and improved colloidal stability in biological environments. Strategies for active targeting using antibodies, peptides, aptamers, and small‐molecule ligands significantly enhance tumor specificity. Furthermore, UiO‐66 is increasingly used as a carrier for photosensitizers, contrast agents, and imaging probes, supporting multimodal fluorescence, CT, MRI, and photoacoustic imaging. The framework's ability to coordinate photosensitizers and modulate oxygen availability provides powerful opportunities for PDT, especially in hypoxic tumors. However, key challenges remain, including long‐term biocompatibility, clearance, and scalable synthesis. Future prospects include programmable degradation, advanced surface architectures, biomimetic coatings, and multimodal phototheranostic platforms.

## Graphical abstract

UiO‐66(Zr) metal–organic frameworks are chemically stable, biocompatible, and highly tunable nanomaterials. Their modular structure enables controlled drug delivery, multimodal bioimaging, and light‐activated photodynamic therapy, supporting integrated diagnostic and therapeutic (theranostic) applications in cancer and biomedical research.

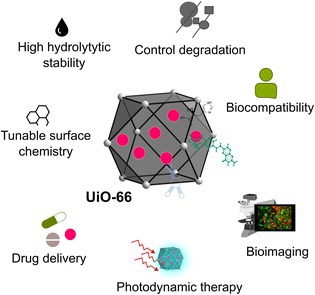

**Abbreviations**
4T1 cells: murine mammary carcinoma cells
AS1411: nucleolin‐binding aptamer
ATG13: autophagy‐related protein 13
ATP: adenosine triphosphate
AuNPs: gold nanoparticles
BAX: Bcl‐2‐associated X protein
Bcl‐2: B‐cell lymphoma 2
BDC: 1,4‐benzenedicarboxylate
Beclin‐1: autophagy‐related protein
BER: berberine
BET: Brunauer–Emmett–Teller
BPDC: bicyclo[1.1.1]pentane‐1,3‐dicarboxylic acid
CCND1: cyclin‐dependent kinase 4
CIS: cisplatin
CT: computed tomography
CT‐26 cells: undifferentiated colon carcinoma cells
DLS: dynamic light scattering
DNA: deoxyribonucleic acid
DOX: doxorubicin
EE: encapsulation efficiency
EPI: epirubicin
FA: folic acid
fcu: face‐centered cubic
FESEM: field emission scanning electron microscopy
GA: glycyrrhetinic acid
GSH: glutathione
HASMC cells: human aortic smooth muscle cells
HEK293A cells: human embryonic kidney 293 cells
HeLa: human cervical cancer cells
HSF cells: human splenic fibroblasts
HUVEC: human umbilical vein endothelial cells
IBU: ibuprofen
ICG: indocyanine green
L929 cells: fibroblast‐like cell line derived from the subcutaneous connective tissue in mouse
LA: lactobionic acid
LC: loading capacity
MCF‐10A: nontumorigenic human breast epithelial cells
MCF‐7/ADR cells: multidrug‐resistant breast cancer cells
MDA‐MB 231: human triple‐negative breast cancer cells
MOF: metal–organic framework
MRI: magnetic resonance imaging
NIR: near‐infrared
OX: oxaliplatin
p53: tumor protein p53
PBS: phosphate‐buffered saline
PDT: photodynamic therapy
PEG: polyethylene glycol
PPIX: protoporphyrin IX
RGD: Arginyl‐Glycyl‐Aspartic acid peptide
ROS: reactive oxygen species
SBU: secondary building unit
SEM: scanning electron microscopy
SERS: surface‐enhanced Raman scattering
TEM: transmission electron microscopy
TPP^+^
: triphenylphosphonium
Tris: tris(hydroxymethyl)aminomethane
UiO: University of Oslo
VEGF: vascular endothelial growth factor
Wi‐38 cells: diploid human fibroblast cell line derived from embryonic lung tissue

Metal–organic frameworks (MOFs) are crystalline porous materials formed by the coordination of metal clusters and organic linkers into ordered architectures with high surface area and tunable chemistry [1]. Their exceptionally high surface area, tunable pore size, and modular composition make them attractive for a broad range of applications, including gas storage [2, 3, 4], catalysis [5, 6, 7], sensing [8, 9, 10], energy storage [11, 12, 13], water remediation [14, 15], and increasingly, biomedical technologies [16, 17, 18, 19]. In the past decade, the intersection of MOF chemistry with biological and medical research has expanded rapidly, driven by the demand for versatile nanomaterials capable of interacting with complex biological environments [20].

However, translation of MOFs into bio‐related applications requires materials that combine high porosity with outstanding chemical and hydrolytic stability, controllable particle size, and low toxicity. Among the various MOF families, zirconium‐based frameworks have emerged as particularly promising candidates due to their robust metal–oxygen bonds and resistance to degradation under physiological conditions [21, 22]. Zirconium (IV)‐based MOFs are constructed from zirconium–oxo coordination nodes interconnected by carboxylate linkers, forming highly stable three‐dimensional networks. The strong Zr–O coordination imparts remarkable thermal and chemical stability, including resistance to water and buffer solutions across a wide *pH* range [23]. These properties are essential for biomedical implementation, as nanomaterials must retain structural integrity in aqueous, protein‐rich, and often mildly acidic physiological environments. Due to these properties, Zr‐MOFs have been positioned as leading platforms for drug delivery [24, 25], imaging [26, 27], biosensing [28, 29], and antimicrobial systems [30, 31, 32].

Among Zr‐based MOFs, UiO‐66 (University of Oslo), first reported in 2008 by Cavka *et al*. [33], is one of the most extensively studied and widely applied structures in biomedical research. UiO‐66 crystallizes in a face‐centered cubic (*fcu*) topology in which [Zr_6_(*μ*_*3*_‐O)_4_(*μ*_*3*_‐OH)_4_(*μ*_*2*_‐COO)_12_] clusters act as 12‐connected secondary building units linked by derivatives of 1,4‐benzenedicarboxylate (BDC) ligands. Each Zr(IV) center exhibits a coordination number of eight and adopts a distorted tetragonal antiprismatic geometry (Fig. 1A). Six Zr atoms are arranged at the vertices of an octahedron, while *μ_3_*‐O and *μ_3_*‐OH ligands cap each triangular face in a triply bridging coordination mode (Fig. 1B). Twelve carboxylate groups originating from 12 terephthalate linkers coordinate to the cluster edges in a *syn–syn* bridging mode (Fig. 1C). This specific coordination scheme results in a highly symmetric secondary building unit with the shape of a cuboctahedron (Fig. 1D), which further assembles into the extended *fcu* framework of UiO‐66. The resulting framework contains tetrahedral (~8 Å) and octahedral (~11–13 Å, Fig. 1E) cages accessible through triangular windows (~6 Å), generating permanent microporosity and a high internal surface area, with typical Brunauer–Emmett–Teller (BET) values ranging from 1000 to 1500·m^2^·g^−1^ depending on synthesis conditions and defect concentration [34, 35]. This coordination environment endows UiO‐66 with hydrolytic, thermal, and mechanical stability, enabling preservation of crystallinity in aqueous and physiological environments and facilitating further processing into nanostructured or composite systems [22, 36]. However, it should be noted that UiO‐66 is hydrolytically stable in pure water but can partially degrade in phosphate‐containing buffers (PBS) and at acidic pH (4–5), conditions relevant for intracellular compartments and tumor microenvironments [37]. Indeed, the combination of uniform pore geometry and high surface area facilitates encapsulation of small molecules, photosensitizers, and biologically active cargos. The advantage of UiO‐66 over other MOFs with biomedical potential is shown in Table 1.

**Fig. 1:**
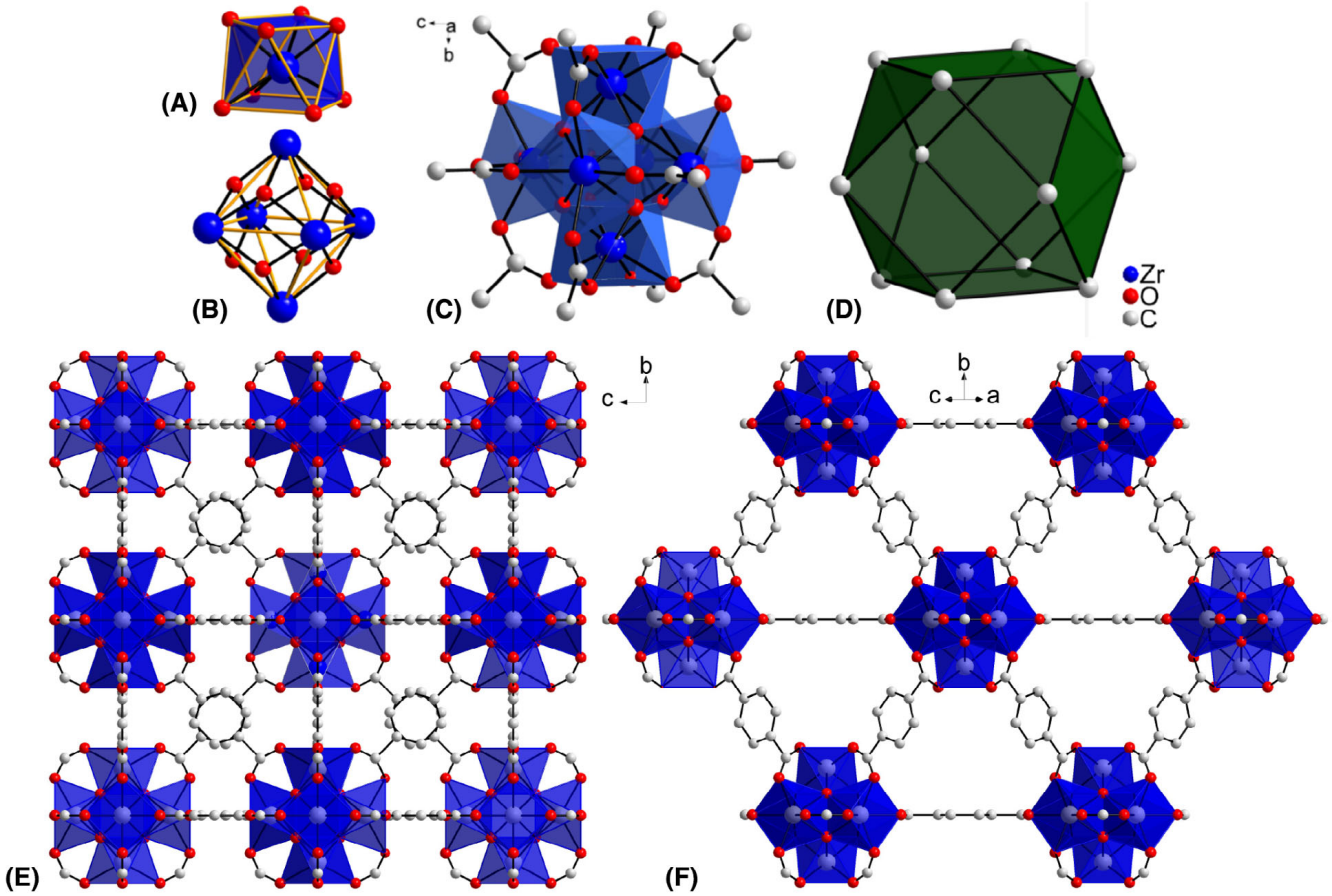
(A) Coordination geometry of the Zr(IV) center exhibiting a distorted tetragonal antiprismatic arrangement. (B) Inorganic [Zr_6_(*μ*_*3*_‐O)_4_(*μ*_*3*_‐OH)_4_] core showing six Zr atoms located at the vertices of an octahedron, with *μ*_*3*_‐O and *μ*_*3*_‐OH ligands capping each triangular face. (C) Cuboctahedral [Zr_6_(*μ*_*3*_‐O)_4_(*μ*_*3*_‐OH)_4_(*μ*_*2*_‐COO)_12_] secondary building unit (SBU) formed by 12 carboxylate groups from terephthalate (BDC) linkers bound in a *syn–syn* bridging mode to the edges of the SBU. (D) Representation of the resulting cuboctahedral SBU assembling into the extended (E) face‐centered cubic (*fcu*) framework of UiO‐66 and (F) accessible triangular windows (~6 Å).

**Table 1:** Comparative physicochemical and biomedical characteristics of UiO and selected metal–organic frameworks families (ZIF, MIL, and HKUST).

MOF		Particle size	Biocompatibility	Drug loading	Pore size	Stability	Key advantages	Main limitations
UiO family [1, 22]	UiO‐66 (Zr) [38, 39]	115.2 (FESEM), 124.3 nm (DLS)	Nontoxic at 1000 μg·mL^−1^ (CT‐26 cells)	LC 29.3%	0.38 nm [40]	Stable, 18% degradation (N_2_ isotherm; 3 m; H_2_O) [41] Highly stable (XRD/SEM/N_2_ isotherm; 100 days; DI water + saline solutions) [42] Stable (PXRD; no change after 24 h, KOAc ≤1 m) [43] Stable, 12% degradation (N_2_ isotherm; 4 h; TRIS, *pH* = 7.5) [44] Unstable, 91% degradation (N_2_ isotherm; 4 h; PBS, *pH* = 7.5) [44]	Exceptional hydrolytic stability Very robust functionalization Reliable particle size control	Limited accessibility for medium‐to‐large cargoes
		90–120 nm (TEM)	75% at 1000 μg·mL^−1^ (HSF cells)	LC 6.26%, EE 19.7%				
	UiO‐67 (Zr) [45, 46]	40 nm (SEM), 100 nm (DLS)	90% at 300 μg·mL^−1^ (4 T1 cells)	LC 82%	0.55 nm [40]	Stable (PXRD; 5 days, PBS) [46]	High hydrolytic stability Robust functionalization	Limited accessibility for large cargoes
		150 nm (DLS)	–	LC 36.5%, EE 19.2%				
ZIF family [47, 48]	ZIF‐8 (Zn) [49, 50]	100 nm (TEM)	75% at 50 μg·mL^−1^ (HeLa cells)	LC 10%	0.34 nm [51]	Stable (intrusion porosimetry; ≤4 days, water) [52] Unstable (Atomic force microscopy; ~15 min, PBS) [53]	pH‐responsive cargo release Reliable particle size control	Limited hydrolytic stability
		80 nm (TEM), 137.5 nm (DLS)	20% at 40 μg·mL^−1^ (MCF‐7/ADR cells)	LC 9.4%, EE 58.2%				
MIL family [54, 55]	MIL‐53 (Fe) [56, 57]	180 nm (DLS)	90% at 450 μg·mL^−1^ (MCF‐7 cells)	LC 43.1%, EE 75.7%	Flexible, 15 nm (N_2_ isotherm) [58]	Partly degrades (SEM; 48 h, Tris) [56]	High hydrolytic stability Breathing flexibility *pH*‐responsive release	Moderate hydrolytic stability Limited accessibility for larger cargoes
		120 nm (SEM)	80% at 200 μg·mL^−1^ (HASMC cells)	EE 28.0%				
	MIL‐100 (Fe) [59, 60, 61]	165 nm (DLS)	94% at 100 μg·mL^−1^ (MCF‐7 cells)	LC 36.4%, EE 28.6%	0.86 nm [51]	Relatively stable in an aqueous solutions (DLS; 24 h, PBS) [61]	High loading capacity	Limited accessibility for large cargos
		165 nm (DLS)	92% at 200 μg·mL^−1^ (MCF‐7 cells)	LC 27.8%, EE 80,0%				
		87.5 (TEM/SEM), 102.8 (DLS)	90% at 200 μg·mL^−1^ (MCF‐7 cells)	LC 28.0%, EE 80%				
	MIL‐101 (Fe) [62, 63, 64]	723 nm (DLS)	85% at 1000 μg·mL^−1^ (L929 cells)	LC 10.1%, EE 25.4%	1.2 nm [65]	40% degradation (12 h, PBS) [62]	Very high loading capacity	Moderate hydrolytic stability
		50 nm (TEM/SEM)	70% at 200 μg·mL^−1^ (MCF‐10A cells)	LC 54.9%, EE 41.6%				
		500 nm (SEM), 458 nm (DLS)	85% at 48 μg·mL^−1^ (Wi‐38 cells)	LC 22.5%, EE 75.1%				
HKUST family	HKUST‐1 (Cu) [66, 67]	25 000 nm (SEM)	82% at 60 μg·mL^−1^ (HEK293A cells)	LC 1.5% (IBU), LC 8.0% (DOX)	0.45 nm [68]	40% degradation (10 h, PBS) [67]	Strong biomolecule interaction (open metal sites) [69]	Limited hydrolytic stability
		207.3 (DLS)	90% at 100 μg·mL^−1^ (4 T1 cells)	LC 2.41%				

Functionalized derivatives such as UiO‐66‐NH_2_ [70, 71], UiO‐66‐COOH [71, 72], and UiO‐66‐NO_2_ [70, 71] can be obtained through direct synthesis using substituted dicarboxylate linkers, introducing reactive groups that modulate hydrophilicity, surface charge, and interactions with biomolecules. Further structural diversification is achieved via postsynthetic modification, including amide coupling, click reactions, or Schiff base formation [73, 74, 75], allowing covalent attachment of drugs, targeting ligands, polymers, fluorescent probes, or enzymes [73, 76, 77, 78] to internal or external framework surfaces. For biomedical applications, particle downsizing is essential to ensure colloidal stability, cellular uptake, and controlled biodistribution [79, 80]. UiO‐66 nanoparticles with sizes ranging from tens to a few hundred nanometers can be prepared using reaction modulation, microwave‐assisted synthesis, or microemulsion methods [81, 82, 83]. Subsequent surface functionalization with polymeric or lipid coatings (e.g., polyethylene glycol, polydopamine, and lipid bilayers) improves dispersibility in physiological media, reduces nonspecific protein adsorption, and prolongs circulation time, while conjugation of antibodies, peptides, or aptamers enables active targeting toward specific cell types [76, 84, 85, 86].

These attributes position UiO‐66 collectively as a highly adaptable nanoplatform for biomedical applications. Recent studies have demonstrated its potential in passive and targeted drug delivery, *pH*‐responsive release, photodiagnostics, and photodynamic therapy (PDT), as well as multimodal imaging modalities integrating fluorescence, magnetic, photoacoustic, and computed tomography (CT) contrast. However, despite rapid progress, key challenges remain, including the need for standardized toxicity evaluation, a better understanding of long‐term fate and biodegradation, and scalable synthesis of homogeneous nanoparticles suitable for translational studies.

This review summarizes the recent advances in designing UiO‐66‐based nanostructures for bioimaging, controlled drug delivery, phototherapeutic applications, and immunomodulation. Key strategies for framework engineering, targeting, and functional integration are highlighted, and emerging evidence regarding biocompatibility, toxicity, and immune interactions is discussed. Finally, current barriers and future research directions required for the translation of UiO‐66‐based systems toward clinical implementation are outlined.

## Biocompatibility, toxicity, and engineering for *in vivo* safety

Biocompatibility and toxicity represent central considerations in the development of UiO‐66‐based nanomaterials for biomedical applications. Although Zr–carboxylate frameworks are generally viewed as chemically robust, their interactions with biological environments are complex and strongly dependent on particle size, surface chemistry, colloidal stability, and the composition of surrounding physiological fluids. A growing frame of evidence indicates that UiO‐66 nanoparticles can exhibit excellent biocompatibility when properly engineered, yet may also undergo structural degradation or induce cellular responses if not adequately stabilized.

### Chemical stability and degradation in physiological conditions

In aqueous environments, UiO‐66 shows higher hydrolytic stability than most MOFs due to the strong Zr–O coordination bonds within the Zr_6_O_4_(OH)_4_ secondary building unit [87]. However, this stability is not fixed. Phosphate‐containing buffers such as PBS can partially displace carboxylate linkers, leading to gradual degradation of the coordination nodes [44]. Notably, phosphate concentrations in PBS (~10 mm) are significantly higher than those found in physiological fluids (~1 mm in plasma), and phosphate ions *in vivo* are often partially bound or compartmentalized, suggesting that PBS may overestimate the extent and rate of MOF degradation under biological conditions. Similarly, mildly acidic microenvironments (*pH* 4–5), characteristic of tumor tissue and lysosomal compartments, promote partial dissociation of Zr–O bonds [88, 89, 90]. While such degradation presents a challenge for long‐term structural persistence, it can be purposefully leveraged for *pH*‐responsive release of therapeutics, enabling selective cargo liberation in tumor or intracellular compartments while maintaining stability under physiological *pH* [89].

### Cellular interactions and cytotoxicity profiles

Cytotoxicity of UiO‐66 nanoparticles is closely coupled to surface functionalization and colloidal behavior. Unmodified particles may adsorb serum proteins, aggregate, or interact nonspecifically with cell membranes, occasionally leading to oxidative stress or reduced cell viability at high concentrations [91, 92, 93]. In contrast, surface engineering strategies, such as PEGylation, coating with polydopamine or biocompatible polymers, or decorating the surface with hydrophilic functional groups, substantially reduce nonspecific interactions and enhance cytocompatibility [94, 95, 96]. Numerous studies demonstrate that appropriately coated UiO‐66 nanoparticles exhibit low toxicity in a wide range of cell lines, even at therapeutically relevant concentrations, underscoring the importance of surface chemistry in determining biocompatibility [97, 98, 99, 100].

### Immune system interactions and immunomodulatory effects

Beyond simple biocompatibility, UiO‐66 nanoparticles can interact with the immune system in ways that are increasingly recognized as relevant for therapeutic design. Their uptake by macrophages and dendritic cells may influence cytokine production, macrophage polarization, or antigen presentation [101, 102]. In some formulations, UiO‐66 has been shown to modulate immune signaling, contributing to anti‐inflammatory or immunostimulatory effects depending on the context and surface chemistry [103, 104]. These interactions hold potential for immunotherapy, vaccine delivery, or adjuvant development, but also emphasize the need for rigorous biological evaluation to avoid unintended immune activation.

Immunostimulation associated with MOF structures is an important biological feature that can be exploited to enhance therapeutic outcomes [105, 106]. For example, UiO‐66(Zr)‐NH_2_ loaded with curcumin and functionalized to target myocardial collagen improved the myocardial microenvironment in mice suffering from inflammation‐associated fibrosis [107]. Additionally, UiO‐66(Zr)‐NH_2_ can co‐deliver immunomodulatory agents, such as rapamycin and interleukin‐1 receptor antagonist, effectively reducing atherosclerotic plaques in major arteries by regulating macrophage activity, cytokine expression, and autophagy pathways [108]. These findings demonstrate that UiO‐66(Zr)‐based delivery systems can be targeted for the treatment of cardiovascular diseases, atherosclerosis, inflammatory disorders, and osteoarthritis through macrophage polarization and reactive oxygen species (ROS) scavenging [107, 108, 109].

### Surface engineering strategies to enhance *in vivo* applicability

To improve systemic safety, circulation time, and biodistribution, a variety of surface modification strategies have been developed: (i) *PEGylation*: Shields nanoparticles from opsonization, reduces protein corona formation, improves colloidal stability, and prolongs blood circulation [76, 100, 110]. (ii) *Polydopamine coatings*: Provide a versatile reactive surface, improve stability in physiological media, and enable further functionalization with targeting ligands or enzymes [85, 111, 112]. (iii) *Biopolymer coatings (e.g., chitosan and hyaluronic acid)*: Enhance mucoadhesion or receptor‐specific uptake, and may impart additional bioactivity or *pH* sensitivity [25, 85, 113, 114]. (iv) *Biomimetic membrane coatings (e.g., red blood cell membranes)*: Camouflage nanoparticles to evade immune recognition and extend systemic circulation [115, 116]. Together, these strategies minimize off‐target interactions, reduce cytotoxicity, and promote more predictable *in vivo* behavior.

A notable example of polymeric surface engineering is the use of hyaluronic acid, a disaccharide‐based biopolymer. When applied alongside polydopamine to UiO‐66(Zr)‐NH_2_, it stabilized curcumin for oral administration in models of ulcerative colitis, a chronic inflammatory bowel disease, where it helped suppress inflammation, modulate intestinal immune responses, and promote macrophage polarization [85]. Hyaluronic‐acid functionalization is also effective for anticancer drug delivery. Recently, daunorubicin was loaded into hyaluronic‐acid‐modified UiO‐66(Zr)‐COOH incorporating a citric‐acid dendrimer, achieving a high loading efficiency (~74%) [114]. This system was degradable in a lysozyme‐rich environment, exhibited *pH*‐responsive behavior, and demonstrated strong anticancer activity against breast cancer cells.

### Biodistribution, clearance, and translational considerations

*In vivo* studies indicate that nanoparticle size, surface charge, and coating determine biodistribution, with unmodified UiO‐66 often accumulating in the liver and spleen due to uptake by the mononuclear phagocyte system [117, 118, 119, 120]. Surface engineering can modulate this profile, enabling improved tumor accumulation or reduced hepatic retention. Despite encouraging results, the long‐term fate, biodegradation products, and clearance mechanisms of UiO‐66 nanoparticles remain insufficiently understood, particularly for repeated dosing or chronic exposure scenarios. Addressing these gaps will be crucial for advancing UiO‐66 platforms into clinical translational pathways.

## Drug delivery in UiO‐66: Passive, targeted, and stimuli‐responsive strategies

The intrinsic porosity, high surface area, and structural robustness of UiO‐66(Zr) make this framework an attractive nanoplatform for therapeutic delivery. Beyond passive encapsulation of small molecules, UiO‐66 nanoparticles can be engineered to achieve controlled cargo release, tumor‐selective accumulation, and enhanced therapeutic efficacy through surface functionalization and integration of responsive motifs. This section summarizes key strategies, including passive loading, *pH*‐ and stimuli‐responsive release, and ligand‐mediated targeting that underpin the growing biomedical utility of UiO‐66‐based systems.

### Passive encapsulation and delivery of small‐molecule therapeutics

Many delivery systems have been designed to exploit the enhanced permeability and retention (EPR) effect, which passively facilitates the accumulation of nanoparticles and macromolecules within tumors owing to their leaky vasculature and insufficient lymphatic drainage [121, 122]. Although passive targeting can improve intratumoral drug levels without requiring additional targeting ligands, the physicochemical properties of most therapeutic molecules, particularly their hydrophobicity, tendency to aggregate, limited stability, and poor bioavailability, often hinder the achievement of sufficiently high concentrations in tumor tissue [123, 124]. The intrinsic microporosity and large internal volume of UiO‐66(Zr) nanoparticles provide a high loading capacity for a broad range of hydrophobic and hydrophilic therapeutics [125, 126], with representative molecules, loading capacities, and encapsulation efficiencies summarized in Tables 1 and 2. Encapsulation within the UiO‐66 framework can substantially mitigate the adverse effects associated with free drugs, including instability, poor solubility, and rapid clearance [127, 128, 129, 130]. Moreover, suitable surface modifications can further prevent premature drug release and enhance the overall bioapplicability and *in vivo* performance of MOF‐based nanocarriers.

**Table 2:** Summary of drug‐loading performance, pore characteristics, and surface‐functionalization strategies in UiO‐66(Zr) nanocarriers. NR, not reported.

Full MOF formula, standardized	Particles' size	Drug	Drug loading	Pore structure	Surface modification	Modification purpose
DOX@FA/TPP^+^‐NH_2_‐UiO‐66 [131]	90–130 nm (TEM)	Doxorubicin (DOX)	LC 6.04 ± 0.49%	BET 850 m^2^·g^−1^; Pore size 0.6–1.4 nm; Pore volume 0.405 cm^3^·g^−1^	Folic acid (FA), triphenylphosphonium (TPP^+^)	Folic acid (FA)—folate receptor‐mediated cancer‐cell targeting Triphenylphosphonium (TPP^+^)—mitochondrial targeting
DOX@LA/GA‐NH_2_‐UiO‐66 [39]	90–120 nm (TEM)	Doxorubicin (DOX)	LC 6.26 ± 0.04%	BET 750 m^2^·g^−1^; Pore size 0.6 and 1.2 nm	Lactobionic acid (LA), glycyrrhetinic acid (GA)	Lactobionic acid (LA)—targeting asialoglycoprotein receptors for hepatocellular carcinoma cells Glycyrrhetinic acid (GA)—GA receptor‐mediated liver cancer targeting
DOX@OCA/LA‐NH_2_‐UiO‐66 [132]	248 ± 7.3 nm (DLS)	Doxorubicin (DOX)	LC 25%; EE 76%	BET 398 m^2^·g^−1^; Pore size ~ 0.6 and 1.2 nm	Lactobionic acid (LA), obeticholic acid (OCA)	Lactobionic acid (LA)—hepatocellular targeting via ASGPR receptor Obeticholic acid (OCA)—nuclear targeting via FXR receptor; also acts as anticancer agent
EPI@UiO‐66@PEG‐FA [133]	30–50 nm (TEM/SEM); 222.8 ± 6.1 nm (DLS)	Epirubicin (EPI)	EE 75.33 ± 1.34%	NR	Polyethylene glycol (PEG), folic acid (FA)	Polyethylene glycol (PEG)—improves circulation stability and reduces nonspecific interactions with plasma proteins/immune cells Folic acid (FA)—tumor targeting via folate receptor overexpressed on cancer cells
OX@FA‐UiO‐66‐NH_2_ [134]	115.15 ± 1.97 nm (FESEM); 297.9 ± 47.6 nm (DLS)	Oxaliplatin (OX)	LC 29,3%	BET 376.14 m^2^·g^−1^; Pore size 0,73 nm; Pore volume 0.2167 cm^3^·g^−1^	Folic acid (FA)	Folic acid (FA)—targeting folate receptors overexpressed on colorectal cancer cells
Ber@NH_2_‐UiO‐66@PEG‐FA [110]	140.49 nm (TEM); 134.31 nm (DLS)	Berberine (Ber)	LC 15.55%	NR	Polyethylene glycol (PEG), folic acid (FA)	Polyethylene glycol (PEG)—improves hydrophilicity, stability and prolongs blood circulation of nanoparticles Folic acid (FA) – active targeting of tumor cells via folate receptors
CIS@FA‐NH_2_‐UiO‐66 [135]	236.2 ± 6.74 nm (DLS)	Cisplatin (CIS)	EE 71.52 ± 1.15%	NR	Folic acid (FA)	Folic acid (FA)—targeting folate receptors overexpressed on cancer cells via receptor‐mediated endocytosis
DOX@UiO‐66‐NH_2_‐FA [38]	124.27 nm (DLS)	Doxorubicin (DOX)	LC 36.25%	BET 323 m^2^·g^−1^; Pore volume 16.7 cm^3^·g^−1^	Folic acid (FA)	Folic acid (FA)—folate receptor targeting of cancer cells
DOX/ICG@UiO‐66@PDA‐TF [136]	100–170 nm (SEM/TEM); 194.8 nm (DLS)	Doxorubicin (DOX); Indocyanine green (ICG)	DOX EE >90%; ICG EE >90%	BET 971.98 m^2^·g^−1^	Polydopamine (PDA), transferrin (TF)	Polydopamine (PDA)—prevent DOX leakage and improve photothermal performance/stability Transferrin (TF)—active tumor targeting via transferrin receptor
TPZ@UiO‐66(Zr/Cu)‐FA [137]	140–160 nm (TEM)	Tirapazamine (TPZ)	–	BET 614.77 m^2^·g^−1^; Pore volume 0.35 cm^3^·g^−1^	Folic acid (FA)	Folic acid (FA)—tumor targeting via folate receptor‐mediated uptake
DOX@UiO‐66@aptATP‐DNAtetra [138]	200–300 nm (SEM/STEM); ~400 nm (DLS)	Doxorubicin (DOX)	LC 65 nmol mg^−1^	Pore size 8 Å and 11 Å	DNA tetrahedra, ATP aptamer	DNA tetrahedra—nanoparticle gating structure and enhanced cellular permeation ATP aptamer—ATP‐triggered unlocking of the nanocarrier and controlled intracellular drug release
DOX@UiO‐66@aptVEGF‐DNAtetra [138]	200–300 nm (SEM/STEM); ~400 nm (DLS)	Doxorubicin (DOX)	LC 71 nmol mg^−1^	Pore size 8 Å and 11 Å	DNA tetrahedra, VEGF aptamer	DNA tetrahedra—nanoparticle gating structure and enhanced cellular permeation VEGF aptamer—VEGF‐triggered unlocking and tumor biomarker‐responsive drug release
Zn(II)‐PPIX@UiO‐66@aptVEGF‐DNAtetra [138]	200–300 nm (SEM/STEM); ~400 nm (DLS)	Zn(II)‐protoporphyrin IX (Zn(II)‐PPIX)	LC 82 nmol mg^−1^	Pore size 8 Å and 11 Å		

### pH‐responsive and stimuli‐triggered drug release

UiO‐66(Zr) is characterized by high stability, but when exposed to acidic conditions and very low *pH*, its porosity and surface area decrease due to partial destruction of its structure, and the surface charge becomes negative, which influences the adsorption capacity of UiO‐66(Zr) [139]. This *pH* sensitivity, rooted in the vulnerability of Zr–O–C coordination bonds under acidic stress, results in decreased surface area, altered surface charge, and accelerated release of encapsulated molecules [140, 141]. However, this effect is highly desirable in cancer treatment, as most tumors have an acidic environment [142]. This property has been advantageously exploited in several therapeutic systems:

#### Curcumin‐loaded UiO‐66(Zr)

Acid‐triggered release in breast cancer cells promoted apoptosis through caspase activation and downregulation of matrix metalloproteinases and cyclin proteins [98]. Biocompatibility and safe delivery of antioxidant compounds, including curcumin, were also observed in liver cancer cells [143]. Improvement of curcumin's biological activity by its loading in UiO‐66 has even been demonstrated in a 3D model of pancreatic cells [144]. Overall, curcumin can be loaded into other MOFs to enhance anticancer activity against various types of cancer [145].

#### Doxorubicin‐loaded UiO‐66‐NH_2_ coated with poly(N‐vinylcaprolactam)

The polymer shell enhanced *pH*‐dependent drug release while improving aerodynamic properties relevant to pulmonary drug delivery [84]. This improved control of doxorubicin release under acidic conditions and increased therapeutic efficacy. Importantly, the UiO‐66(Zr)‐NH_2_ structure provides a consistent platform with controlled aerodynamics, which is important for orotracheal administration in lung drug delivery [101]. Combined with high biocompatibility and *pH*‐controlled drug release, these structures are very attractive.

#### Histidine modified UiO‐66‐NH_2_

His‐functionalization enabled *pH*‐responsive delivery of 5‐fluorouracil via transporter‐mediated uptake, inducing intracellular acidification, autophagy modulation, and apoptosis in glioblastoma models [146]. These modifications allowed UiO‐66(Zr)‐His to target the L‐type amino acid transporter 1, facilitating selective transport of 5‐fluorouracil and resulting in autophagy in healthy dermal cells and apoptosis in glioblastoma spheroids, accompanied by increased intracellular acidification. The biocompatibility and modular architecture of UiO‐66‐based materials further highlight their potential for dual‐drug loading or incorporation of targeting ligands to enhance cancer‐cell specificity.

#### PEG‐coated UiO‐66(Zr)

PEGylation improved biocompatibility and prevented premature vincristine release while enabling pH‐responsive delivery that modulated apoptosis‐related gene expression (BAX, BCL2, p53, CCND1, and CDK4) [100].

These examples underscore how intrinsic *pH* sensitivity and tailored surface chemistry synergize to enhance drug stability in circulation while ensuring effective release within the acidic tumor microenvironment.

External stimuli may further augment release kinetics. Ultrasound‐triggered systems, for example, have been used with UiO‐66‐NH_2_ to improve temozolomide diffusion through the framework, accelerate dissolution, and synergistically boost antitumor activity [147]. Ultrasound also enhances intracellular ROS generation, contributing to combined chemo‐ and sonodynamic therapeutic effects [137].

## Targeted delivery using UiO‐66: Aptamers, transferrin, folic acid, and dual‐ligand architectures

Active targeting represents a crucial strategy for improving the therapeutic selectivity and cellular uptake of nanocarriers. In UiO‐66(Zr)‐based systems, surface modification with biomolecular ligands markedly enhances affinity toward tumor‐associated receptors and enables controlled, receptor‐specific intracellular transport. Several classes of targeting units (including aptamers, transferrin, folic acid, and dual‐ligand combinations) have been successfully integrated into UiO‐66 nanoparticles, each offering complementary advantages for precision drug delivery. Various surface‐functionalization strategies in UiO‐66(Zr) nanocarriers and their performance are summarized in Table 2.

**Fig. 2:**
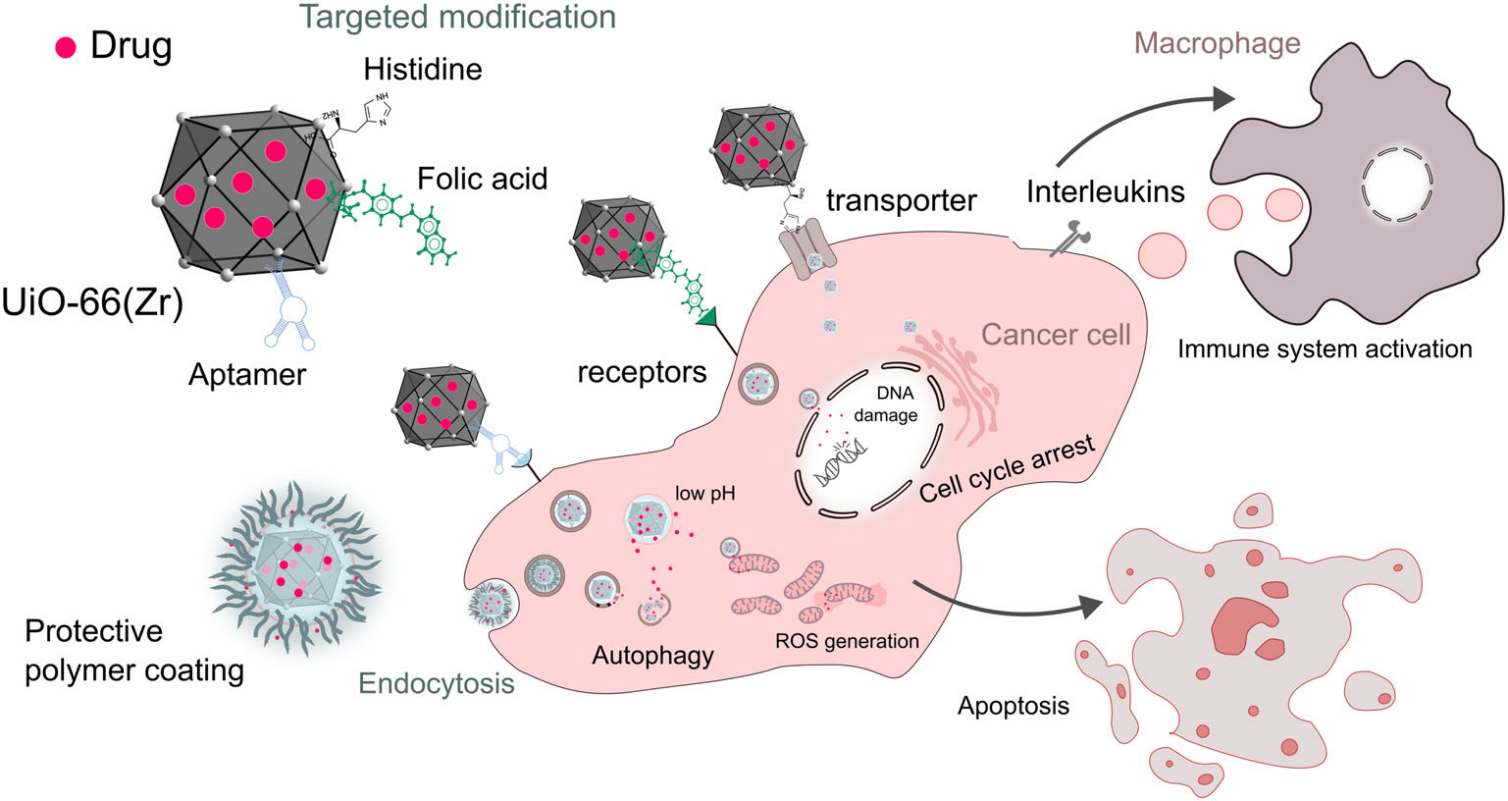
Schematic representation of targeted drug delivery to cancer cells in functionalized UiO‐66(Zr) nanoparticles (aptamer, folic acid, histidine, and polymer coating) and the cellular responses (autophagy, apoptosis, and immune system activation) to potential treatment.

The importance of targeted transport is particularly evident in cancer therapy, where active targeting can increase intratumoral drug accumulation several‐fold compared with healthy tissue [148, 149]. Surface functionalization of nanoparticles with antibodies (e.g., Herceptin), peptides (e.g., RGD), proteins (e.g., transferrin), aptamers, or small molecules (e.g., folic acid and galactose) enables highly specific localization through high‐affinity interactions with overexpressed cell‐surface receptors [150, 151, 152, 153, 154, 155, 156]. Accordingly, nanoparticle design can be tailored to receptors, such as human epidermal growth factor receptor 2 in breast cancer, transferrin receptors in bone marrow, vascular endothelial growth factor receptors in tumor vasculature, and folate or asialoglycoprotein receptors in various cancer cells [157]. Several representative strategies for UiO‐66(Zr) surface functionalization aimed at targeted drug delivery are summarized in Fig. 2.

## Aptamer modification—Targeting cancer biomarkers and designing nanoparticle‐based Aptasensors

Aptamers are oligonucleotides (highly versatile functional nucleic acids) that exhibit excellent stability, facile chemical modification, and strong specificity toward their molecular targets [158]. When integrated with nanomaterials, aptamers enable a new generation of point‐of‐care diagnostic and therapeutic platforms due to the inherent multimodality of nanoparticle systems [159]. In UiO‐66(Zr)‐based nanostructures, aptamers can serve as targeting ligands, gating elements, and sensing components, thereby expanding the capabilities of these MOFs in precision medicine and bioimaging.

A representative example is the design of core–shell hybrid nanoparticles comprising a magnetic core and a UiO‐66‐based shell functionalized with a nucleolin‐binding aptamer [86]. Doxorubicin loaded into these hybrid carriers was selectively delivered to nucleolin‐overexpressing MDA‐MB‐231 triple‐negative breast cancer cells, while uptake by normal HUVEC cells remained minimal. The aptamer simultaneously acted as a pore‐closing gate, opening only upon interaction with nucleolin. The intrinsic *pH* sensitivity of aminated UiO‐66(Zr), which undergoes protonation and partial degradation under acidic conditions, further enabled controlled drug release within the acidic tumor microenvironment. Conjugation with fluorescent carbon dots enhanced imaging capabilities, resulting in a multifunctional nanosystem suitable for magnetically guided, targeted drug delivery.

Beyond cancer therapy, aptamer‐functionalized UiO‐66 derivatives have been employed in advanced sensing platforms. Dual modification of UiO‐66(Zr) with a *β*‐lactoglobulin‐binding aptamer and tetrakis(4‐carboxyphenyl)porphyrin enabled a fluorescence and peroxidase‐mimetic biosensor for allergen detection in food matrices [160]. Similarly, Cu‐doped UiO‐66(Zr) postsynthetically modified with maleimide groups and aptamers produced a highly sensitive smart probe capable of simultaneous detection and intracellular imaging of glutathione and ATP [161].

Aptamers also facilitate controlled cargo release. UiO‐66(Zr) nanoparticles gated with DNA tetrahedra functionalized with aptamers responsive to adenosine triphosphate or vascular endothelial growth factor allowed selective intracellular release of doxorubicin or zinc protoporphyrin, an established photosensitizer used in PDT [138]. These nanosystems induced pronounced cytotoxicity in MDA‐MB‐231 cancer cells, while sparing nonmalignant MCF‐10A cells.

While most aptamer‐modified UiO‐66 platforms rely on fluorescence readouts, recent approaches have explored surface‐enhanced Raman scattering (SERS) to overcome limitations, such as photobleaching [162]. UiO‐66(Zr) nanoparticles gated with tetracycline‐binding aptamers and loaded with methylene blue and gold nanoparticles enabled highly sensitive SERS‐based antibiotic detection in food samples, such as milk and meat [163]. The same SERS‐guided imaging principles were applied to analyze responses of patient‐derived colorectal cancer organoids treated with 5‐fluorouracil delivered via UiO‐66(Zr)‐NH_2_, revealing biomolecular alterations in lipids, proteins, collagen, and DNA using multimodal microscopy techniques [164].

Collectively, these examples (summarized in Table 3) highlight UiO‐66(Zr) as a versatile platform for integrating aptamer‐based targeting, sensing, and controlled release. Its high surface area, tunable porosity, and compatibility with optical, magnetic, CT, and multimodal imaging modalities make aptamer‐functionalized UiO‐66 nanostructures exceptionally promising for next‐generation diagnostic and therapeutic applications [165].

**Table 3:** Summary of aptamer‐modified UiO‐66(Zr) systems, detailing the target biomarkers, aptamer conjugation strategies, aptamer‐MOF architecture, and the resulting applications in selective drug delivery, stimuli‐responsive release, and advanced sensing technologies.

Aptamer type	Target biomarker/Analyte	Conjugation chemistry/integration approach	Aptamer‐MOF structural role	Application
AS1411 (nucleolin‐binding aptamer) [86]	Nucleolin overexpressed in MDA‐MB‐231 TNBC cells	Covalent attachment onto UiO‐66 shell grown on magnetic Fe_3_O_4_ core; aptamer acts as ‘gatekeeper’	Aptamer forms a pore‐closing cap that opens upon nucleolin recognition	Targeted DOX delivery; selective uptake; pH‐responsive release; fluorescent imaging enhanced by carbon dots
*β*‐lactoglobulin aptamer [160]	Food allergen *β*‐lactoglobulin	Dual modification of UiO‐66 with aptamer absorbed + TCPP (porphyrin) through surface functionalization	Aptamer is used as a recognition interface	Multimodal sensing: fluorescence + peroxidase‐mimetic biosensing
Thiolate/maleimide‐linked aptamers sensing ATP and glutathione [161]	ATP and glutathione (GSH)	Cu‐doped UiO‐66 postsynthetically modified with maleimide groups enabling covalent aptamer attachment	Aptamer acts as a smart responsive probe for intracellular metabolites	Dual intracellular sensing and real‐time imaging
ATP‐responsive aptamer [138]	ATP abundant in cancer‐cell cytosol	DNA tetrahedron constructed with ATP‐binding aptamer; anchored to UiO‐66 surface forming a molecular gate	Aptamer embedded inside DNA‐tetrahedral gate; acts as a stimuli‐responsive nanovalve	ATP‐triggered release of doxorubicin; selective killing of MDA‐MB‐231 while sparing MCF‐10A
VEGF‐responsive aptamer [138]	VEGF secreted in tumor microenvironment	DNA tetrahedron functionalized with VEGF‐recognizing aptamer; assembled as gating system for UiO‐66	Aptamer controls VEGF‐triggered release of zinc protoporphyrin photosensitizer	Targeted PDT through controlled release of Zn(II)‐PPIX; high specificity to cancer cells
Tetracycline‐binding aptamer [163]	Tetracycline antibiotic	Aptamer‐gated UiO‐66‐NH_2_ loaded with methylene blue and decorated with AuNPs (SERS enhancement)	Aptamer acts as a release regulator + recognition element	SERS‐guided antibiotic detection in food samples (milk, meat)

### Transferrin modification—targeting and prolonged circulation of UiO‐66(Zr) nanoparticles

Transferrin, a glycoprotein involved in iron transport, binds its cognate receptor, which is frequently overexpressed in malignant tissues. Transferrin functionalization thus provides a biologically grounded route for receptor‐mediated targeting and prolonged systemic circulation [166, 167].

Recent work demonstrates that transferrin‐modified UiO‐66(Zr) nanoparticles carrying doxorubicin and indocyanine green form a multimodal platform integrating chemotherapy, photothermal therapy, and PDT [136]. Upon 808 nm irradiation, the system produced robust tumor ablation due to synergistic thermal and ROS‐mediated effects. Additionally, a hierarchical UiO‐66(Zr) system incorporating the near‐infrared (NIR) dye cypate and functionalized with transferrin and polyethylene glycol has been shown to enable targeted multimodal imaging‐guided photothermal and PDT in tumor‐bearing mice, demonstrating strong potential for advanced bioapplications [168].

These findings highlight transferrin as a powerful ligand for deep‐tissue targeting and for augmenting the phototherapeutic performance of UiO‐66‐based nanoplatforms.

### Folic acid modification—Folate receptor targeting to improve diagnostic and treatment of cancer

Folic acid is an essential nutrient required for cell division and metabolic processes [169]. This small molecule binds with high affinity to folate receptors on the cell surface [170], which are overexpressed in many cancers, including colorectal, ovarian, lung, and breast tumors [171, 172, 173, 174]. Conjugation of folic acid to UiO‐66(Zr) nanoparticles enables receptor‐mediated targeting and facilitates the delivery of high drug doses encapsulated within the MOF pores. Folic acid‐modified UiO‐66‐NH_2_ nanoparticles have successfully delivered multiple chemotherapeutics with enhanced potency and reduced off‐target toxicity.

In breast and ovarian cancer cells, treatment with cisplatin loaded into folic acid‐functionalized UiO‐66(Zr)‐NH_2_ led to upregulation of BAX and P53 and downregulation of BCL2, CCND1, and CDK4, indicating targeted delivery, ROS generation, and induction of apoptosis [135]. Similarly, oxaliplatin‐loaded, folate‐modified nanoparticles inhibited the growth of colorectal cancer spheroids more effectively than free oxaliplatin, suppressed cell migration, and induced stronger oxidative stress and apoptosis [134].

Activation of both intrinsic and extrinsic apoptotic pathways was observed in breast cancer cells following folate‐targeted delivery of epirubicin loaded into polyethylene glycol modified UiO‐66(Zr), evidenced by decreased matrix metalloproteinases and increased expression of caspase‐3, caspase‐9, and mitofusin‐1 [133]. The same strategy was applied to oral squamous cell carcinoma using berberine as the therapeutic cargo, resulting in glutathione depletion, ROS generation, and induction of autophagy and apoptosis through upregulation of Beclin‐1, ATG13, BAX, and Bcl‐2, ultimately inhibiting tumor growth [110].

Autophagy can serve as a protective cell‐repair mechanism under certain conditions [175, 176], and its inhibition is therefore a promising approach to shift cell fate toward apoptosis [177]. This concept was utilized in colorectal cancer, where hydroxychloroquine, an autophagy inhibitor, was co‐delivered with doxorubicin using folic acid‐modified UiO‐66(Zr)‐NH_2_, allowing dose reduction while maintaining therapeutic efficacy [38].

Recently, Cu(II)‐doped UiO‐66(Zr) nanoparticles functionalized with folic acid and loaded with tirapazamine were introduced as a dual‐modality system [137]. In this design, folic acid enables receptor‐mediated targeting, while intracellular glutathione reduces Cu(II) ions via a Fenton‐like reaction to generate hydroxyl radicals. Under hypoxic conditions, tirapazamine produces additional hydroxyl and benzotriazinyl radicals, inducing DNA damage and topoisomerase II toxicity. Ultrasound irradiation further amplified radical generation, leading to pronounced cytotoxicity in HeLa cells. The system demonstrated strong anticancer efficacy in a mouse cervical cancer model and provided added benefits for CT imaging and guided hypoxia‐activated therapy.

Multifunctional targeting can be achieved through dual‐ligand surface modification. Nanoparticles decorated with both folic acid and the mitotropic triphenylphosphonium cation enabled mitochondrial targeting, enhancing the apoptotic activity of doxorubicin and inducing cell‐cycle arrest [131]. Similarly, UiO‐66(Zr)‐NH_2_ modified with combinations of folic acid, lactobionic acid, obeticholic acid, or glycyrrhetinic acid exhibited significantly increased cytotoxicity toward hepatocellular carcinoma, demonstrating the potential of dual‐ligand engineering to improve anticancer efficacy [39, 132].

Overall, folic acid functionalization establishes UiO‐66(Zr) nanoparticles as highly selective, receptor‐targeted platforms capable of enhancing diagnostic precision and significantly improving the therapeutic efficacy of diverse anticancer agents.

## Tuning of UiO‐66(Zr) for bioimaging

The integration of imaging functionalities into MOFs has enabled UiO‐66(Zr) based nanoparticles to serve as versatile platforms for diagnostic and theranostic applications. Their high porosity, tunable surface chemistry, and capacity for incorporating optical, magnetic, or radiological agents provide unique opportunities for enhancing imaging sensitivity, spatial resolution, and multimodal contrast in complex biological environments [178].

UiO‐66(Zr) derivatives exhibit intrinsic fluorescence, particularly the amino functionalized UiO‐66‐NH_2_, which displays detectable emission that can be further enhanced by covalent modification with fluorescent moieties [146]. Folic acid functionalization additionally amplifies fluorescence signals [156], while conjugation of 5‐carboxyfluorescein enables real‐time monitoring of targeted delivery of 5‐fluorouracil to tumor tissue [179]. These features illustrate how the structural engineering of UiO‐66 can readily yield efficient optical imaging probes.

The substantial pore volume of UiO‐66 allows simultaneous incorporation of diverse contrast agents, permitting the development of multimodal imaging systems. For X‐ray CT, heavy‐atom doping has proven particularly effective. Hf(IV) incorporation into UiO‐66‐NH_2_, along with iodine loading, considerably enhances CT contrast and enables *in vivo* tumor visualization with high clarity [180, 181]. For magnetic resonance imaging (MRI), paramagnetic components such as cobalt ferrite have been integrated into hybrid UiO‐66‐NH_2_ constructs, producing platforms capable of both MRI contrast generation and photothermal therapy when combined with polydopamine coatings [182].

Near‐infrared photoacoustic imaging represents another rapidly advancing modality enabled by UiO‐66‐based systems [183]. Incorporation of NIR activatable dyes into the framework, or doping with components that enhance photothermal conversion, yields nanoparticles suitable for deep‐tissue visualization and photoacoustic guided therapy. These constructs leverage the deeper penetration and lower tissue scattering of NIR light to achieve improved diagnostic performance and have demonstrated utility even under hypoxic tumor conditions [184, 185].

Photoacoustic imaging, which merges optical excitation with ultrasound detection, offers high‐resolution visualization at millimeter depths. When combined with Raman imaging and CT, UiO‐66 nanoparticles support multimodal, label‐free morpho‐molecular analysis of biological tissues. This combined imaging approach has been applied to patient‐derived colorectal cancer organoids treated with UiO‐66‐NH_2_ delivering 5‐fluorouracil, where complementary optical coherence tomography, multiphoton microscopy, and Raman spectroscopy revealed drug‐induced biochemical alterations in lipids, proteins, collagen, and DNA [164].

**Fig. 3:**
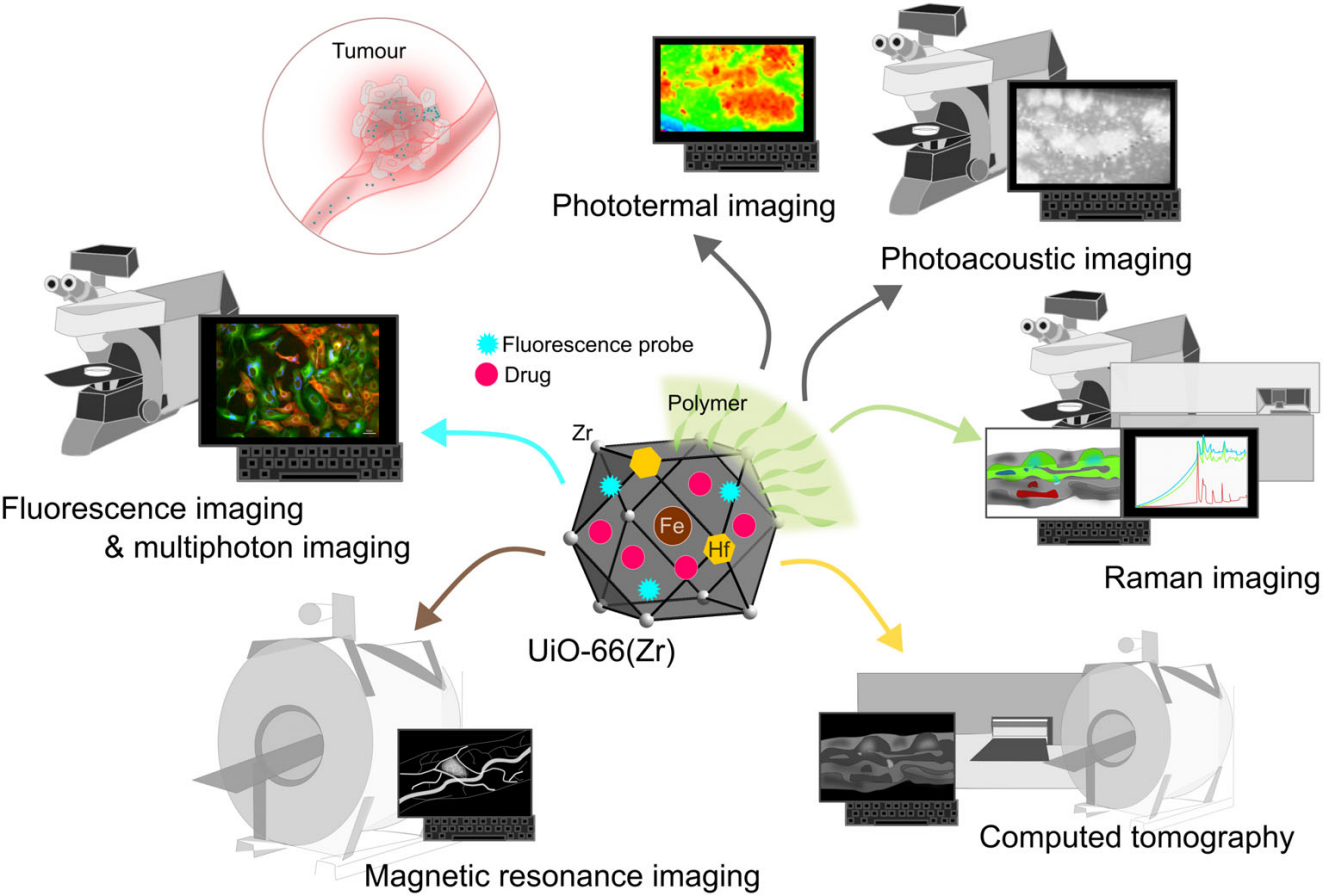
Multimodal approaches for tissue evaluation in which UiO‐66(Zr) nanoparticles are used as contrast agents or modified to enhance contrast and probe delivery.

Overall, UiO‐66‐based materials offer an adaptable scaffold for integrating fluorophores, dyes, magnetic components, and high‐Z elements, enabling flexible design of multimodal imaging probes. Their tunable emission properties, structural robustness, and broad compatibility with molecular contrast agents position UiO‐66 as a powerful platform for advanced diagnostic applications, particularly in cancer imaging, where sensitivity, multiplexing, and deep‐tissue capability are essential. The multimodality of UiO‐66(Zr) achieved through specific modification during synthesis or postsynthesis and its application in bioimaging techniques is summarized in Fig. 3.

## Application of UiO‐66(Zr) nanoparticles in the photodynamic therapy

Photodynamic therapy is a medical treatment with minimal invasiveness and side effects [186]. This modality employs a photosensitizer, a molecule highly sensitive to light, which, in the presence of oxygen, generates ROS toxic to surrounding tissue [187]. The photosensitizer is activated by light whose spectral properties overlap with its absorption, and due to tissue pigment absorption, the most favorable wavelengths are in the red and NIR regions [188, 189].

**Fig. 4:**
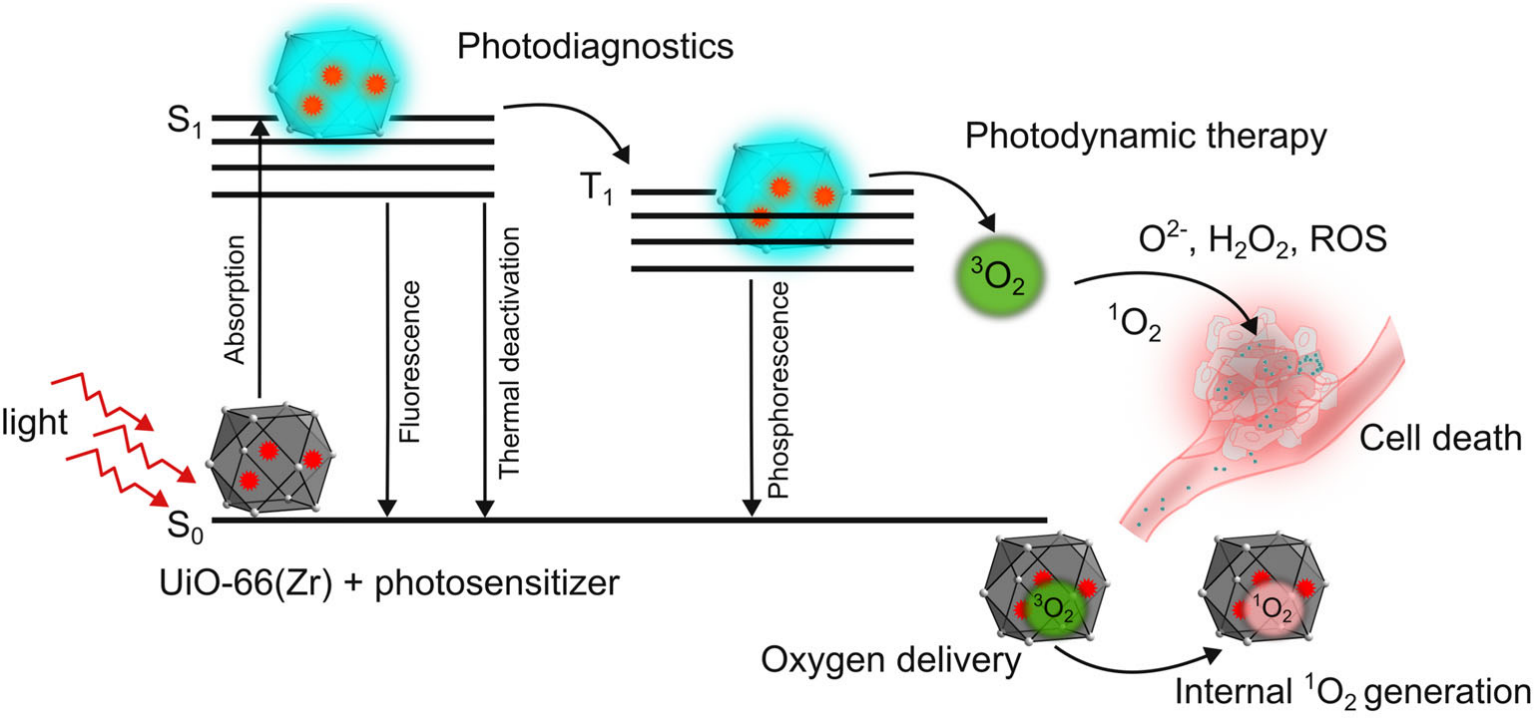
Principle of photodiagnostics and photodynamic therapy using UiO‐66(Zr) as a source of light‐generated reactive oxygen species and for photosensitizer and oxygen delivery.

Incorporating photosensitizers into MOFs can add another degree of multimodality (Fig. 4) to the delivery system, as it can be used in photodiagnostics thanks to the fluorescence of the photosensitizer and subsequently for PDT by changing the excitation wavelength to generate oxidative stress [190].

UiO‐66(Zr) provides a robust and modular framework ideally suited for incorporating photosensitizers, co‐delivering therapeutic agents, and enabling multimodal theranostic functions. The porous architecture allows encapsulation or covalent incorporation of porphyrins, chlorins, and other photoactive molecules, while the exceptional chemical stability of UiO‐66 supports controlled ROS generation and reproducible photochemical behavior.

The integration of photosensitizers into UiO‐66 offers unique opportunities for enhancing PDT performance. Systems incorporating porphyrinic or photoswitchable molecules into the UiO‐66 scaffold enable not only stable light‐activated ROS production but also dynamic modulation of singlet oxygen output.

A notable example includes UiO‐66 nanoparticles loaded with two complementary photosensitive molecules: tetrakis(4‐carboxyphenyl)porphyrin and a photoswitchable dithienylethene derivative, which allow reversible control of singlet oxygen generation within cancer cells using specific light triggers [191]. Such photoswitchable nanoplatforms highlight the potential for externally regulated, on‐demand phototoxicity with high precision.

Hafnium‐doped UiO‐66 nanoparticles loaded with tetratopic chlorins have demonstrated multifunctionality, serving simultaneously as CT contrast agents and as photosensitizer carriers for PDT [192]. Upon 635 nm irradiation, these nanoparticles drastically reduced tumor volume in mouse models following intratumoral injection, illustrating the synergy of heavy‐metal doping and MOF‐based photosensitizer delivery.

### Targeted and multifunctional PDT Nanoplatforms

Surface functionalization further enhances the selectivity and therapeutic efficacy of UiO‐66‐based PDT systems. Pemetrexed‐decorated UiO‐66 nanoparticles serve as an example, using folate receptor targeting to increase nanoparticle accumulation in receptor‐positive tumors [193]. Co‐incorporation of aminolevulinic acid and fluorescent labels enables combined fluorescence imaging and PDT within a single construct.

The porous nature of UiO‐66 also supports multidrug co‐delivery strategies. Systems co‐loaded with doxorubicin, camptothecin, and rhodamine B within folic acid‐modified UiO‐66 nanoparticles achieved synergistic chemotherapy‐PDT effects, activating singlet oxygen production upon 552 nm irradiation and inducing apoptosis through complementary molecular pathways [194].

Advances in hybrid nanostructures have further expanded functionality. UiO‐66‐NH_2_ decorated with upconverting nanorods forms a core‐satellite structure in which NIR excitation of the nanorods produces visible emission that activates porphyrin‐based photosensitizers, enabling deep‐tissue PDT [195]. Simultaneous *pH*‐responsive release of 5‐fluorouracil enabled combined photodynamic‐chemotherapeutic activity with potent cytotoxic outcomes *in vitro*.

### Overcoming tumor hypoxia in PDT

Hypoxia is a major barrier to effective PDT, as oxygen scarcity restricts ROS generation. UiO‐66‐based designs address this challenge either by exploiting hypoxia‐activated prodrugs, supplying oxygen, or reducing intracellular oxygen levels to enhance prodrug activation.

One strategy couples tirapazamine, a hypoxia‐activated cytotoxin, with chlorin e6 within UiO‐66 nanoparticles [196]. The system generates singlet oxygen under 660 nm light while simultaneously activating tirapazamine under hypoxic conditions, enabling dual‐mode cytotoxicity. Another approach employs polydopamine‐coated UiO‐66 nanoparticles loaded with tirapazamine and perfluorotributylamine, an oxygen‐absorbing agent [111]. Under 808 nm irradiation, polydopamine produces a photothermal effect that locally consumes oxygen, thereby amplifying tirapazamine activation and inducing apoptosis in osteosarcoma cells.

Conversely, UiO‐66 can also act as an oxygen reservoir. Oxygen‐loaded UiO‐66(Zr) nanoparticles coated with red blood cell membranes have been used to enhance indocyanine green‐mediated PDT in hypoxic breast cancers [115]. The biomimetic coating promotes immune evasion and passive tumor accumulation, while stored oxygen boosts singlet oxygen generation under 808 nm irradiation, significantly inhibiting tumor growth *in vivo*.

Together, these advances highlight UiO‐66(Zr) as a highly adaptable platform that enhances multiple dimensions of PDT, including targeted photosensitizer delivery, deep‐tissue light activation, and improved therapeutic performance under hypoxic tumor conditions.

## Prospects and future direction of UiO‐66 nanoparticle design

UiO‐66 has established itself as one of the most versatile and chemically robust MOF platforms for biomedical research, yet several important scientific and translational challenges must be addressed to realize its full therapeutic potential. While great progress has been made in leveraging its permanent porosity, structural stability, and modular surface chemistry for applications in drug delivery, bioimaging, and PDT, further developments will be essential to bridge the gap between preclinical proof of concept studies and clinically deployable nanomedicine [22, 197, 198].

A primary challenge lies in achieving a deeper understanding of long‐term *in vivo* fate, biodegradation, and clearance. Despite the high stability of Zr–O coordination bonds, UiO‐66 undergoes partial degradation in acidic environments and in the presence of competing ligands, which is advantageous for controlled drug release but complicates the prediction of long‐term pharmacokinetics [44, 87, 88, 89, 90]. The need for standardized toxicological evaluation is explicitly recognized, as the long‐term *in vivo* behavior of UiO‐66 nanoparticles (including accumulation, immune activation, and clearance pathways) remains incompletely characterized [97, 98, 99, 100, 117, 118, 119, 120]. Future studies should therefore focus on multi‐organ biodistribution, chronic toxicity, and protein corona dynamics under physiologically relevant conditions.

Another key limitation is the scalability and reproducibility of UiO‐66 nanoparticle synthesis. Producing uniform particles with controlled defect density, consistent surface functionality, and stable colloidal properties is critical for regulatory approval and future clinical translation. Greener, scalable synthesis routes and improved control over particle homogeneity have therefore become essential priorities in the field [70, 71, 72, 81, 82, 83].

Emerging therapeutic directions further underscore the importance of advanced surface engineering. Next‐generation UiO‐66 platforms will likely require programmable degradation profiles, enabling controlled disassembly in response to disease‐specific biochemical cues. Additionally, sophisticated surface architectures incorporating stealth coatings, targeting ligands, and stimuli‐responsive elements are expected to enhance circulation time, cellular uptake, and microenvironment‐specific drug release [74, 75, 76, 94, 95].

Beyond oncology, promising opportunities are emerging in antibacterial, anti‐inflammatory, and cardiovascular applications, particularly in strategies leveraging ROS generation, immunomodulation, or targeted delivery to inflamed tissues. UiO‐66‐based systems have demonstrated the ability to modulate macrophage polarization, enhance antibacterial action, and alleviate inflammatory or cardiovascular disease symptoms [107, 108, 109, 199, 200, 201].

In the field of PDT, maintaining photosensitizer stability, maximizing ROS production, and achieving robust therapeutic performance in hypoxic tumor microenvironments remain central challenges. Integrating next‐generation photosensitizers and combining PDT with synergistic therapeutic modalities such as photothermal and ultrasound‐responsive treatments represents a promising direction toward enhanced phototheranostic efficacy [123, 124, 125, 126, 128, 130, 137, 146, 147].

Overall, future advances in UiO‐66 nanomedicine will rely on the combined progress in rational framework design, scalable synthesis, mechanistic toxicology, and disease‐specific engineering. Continued interdisciplinary work, including materials chemistry, nanomedicine, imaging science, and translational biology, will be essential to establish UiO‐66(Zr) as a clinically viable platform for next‐generation phototheranostic and targeted therapeutic applications [100, 148, 149, 150, 151, 152, 153, 154].

## Conclusion

UiO‐66 has emerged as a robust and highly adaptable platform for biomedical applications, offering permanent porosity, structural stability, and modular functionalization suitable for drug delivery, bioimaging, and light‐activated therapies. Advances in nanoparticle engineering now allow precise control over surface chemistry and cargo loading, enabling improved colloidal stability, targeted uptake, and enhanced photodiagnostic and photodynamic performance.

Despite these achievements, several challenges must be overcome to support clinical translation. A deeper understanding of long‐term biological fate, standardized toxicological evaluation, and scalable production of uniform nanoparticles remains essential. In PDT, maintaining photosensitizer stability and achieving consistent therapeutic outcomes in complex biological environments also requires further refinement.

Future progress will likely focus on programmable degradation, greener synthesis routes, and more sophisticated surface architectures integrating stealth coatings, targeting ligands, and stimuli‐responsive elements. The incorporation of next‐generation photosensitizers and multimodal therapeutic strategies is expected to strengthen the theranostic potential of UiO‐66. Continued interdisciplinary research will be key to establishing UiO‐66 as a benchmark platform for next‐generation nanomedicine.

## Conflict of interest

The authors declare no conflict of interest.

## Author contributions

VH, GR, and MA collected the data and wrote the paper.

## Use of generative AI

AI‐based tools were used solely to assist with language editing and improving clarity and readability of the manuscript text. No AI tools were used for data generation, data analysis, interpretation of results, or figure creation. All scientific content, interpretations, and conclusions remain the responsibility of the authors.

## Data Availability

Data sharing is not applicable to this article as no new data were created or analyzed in this study.
